# Nail tattooing: a novel and minimally invasive technique for enhancing drug penetration through the nail

**DOI:** 10.1007/s13346-026-02102-z

**Published:** 2026-03-26

**Authors:** Carlos Bendicho-Lavilla, Victoria Díaz-Tomé, Iria Seoane-Viaño, Vinicius de-Monte-Vidal, Francisco J. Otero-Espinar

**Affiliations:** Department of Pharmacology, Pharmacy and Pharmaceutical Technology, Paraquasil Group (GI-2109), Faculty of Pharmacy, iMATUS, and Health Research Institute of Santiago de Compostela (IDIS), University of Santiago de Compostela (USC), Santiago de Compostela, 15782 Spain

**Keywords:** Nail drug delivery, Tattoo-assisted permeation, Onychomycosis treatment, Transungual drug transport, Ciclopirox lacquer

## Abstract

Onychomycosis, a prevalent fungal infection, and psoriasis, a chronic immune disorder affecting the nail plate, present therapeutic challenges due to the limited efficacy of current treatments, often leading to prolonged therapy and a high relapse rate. The highly cross-linked keratin network of the nail plate acts as a barrier, impeding effective drug delivery. This pioneering study explores a novel approach using a tattoo device to enhance drug penetration through the nail. Ciclopirox olamine and clobetasol lacquers were selected as the model formulations. Drug permeation tests conducted on non-treated, filed (mechanically abraded), and tattooed nails demonstrated significantly higher drug permeation in tattooed nails, suggesting the potential of this delivery method. Nail tattooing offers a simple method to enhance topical therapy, allowing treatment initiation in the clinic and continuation at home.

## Introduction

Onychomycosis is the most prevalent nail disorder worldwide, responsible for up to 90% of toenail infections and 50% of fingernail infections. This pathology causes nail discoloration, thickening, brittleness, and onycholysis [1], leading not only to pain and functional impairment but also to social and psychological burden [2]. Onychomycosis is particularly common in elderly patients and individuals with comorbidities such as diabetes, psoriasis, or HIV [3, 4]. While dermatophytes are the main pathogens, yeasts and non-dermatophyte molds also contribute [5, 6]. Current treatments present challenges as oral antifungals are effective but limited by systemic toxicity, whereas topical agents are safer but fail to adequately permeate the dense, keratinised nail plate, resulting in prolonged therapy and frequent relapse [7]. The limited efficacy of topical agents, combined with the inappropriate use of oral antifungals, contributes to the emergence of resistant onychomycosis strains, exemplified by terbinafine resistance [8].

The nail barrier is composed of densely cross-linked keratin layers with narrow aqueous pores, strongly restricting molecular transport. Variations in keratin fibre organisation and sulfhydryl crosslinking across these layers influence drug permeation, while cysteine-rich proteins create small pores and channels within the keratin network that further affect transport [7]. Drug permeation is also modulated by nail hydration, thickness, molecular properties, and formulation attributes. To overcome these obstacles, permeation-enhancing strategies have been explored, including abrasion, microneedles, iontophoresis, ultrasound and laser poration [9–16].

Medical tattooing, a minimally invasive and widely practiced procedure, has been safely used for scar camouflage, alopecia, and nipple-areolar pigmentation [17]. Beyond cosmetic use, it has also been explored for dermal drug delivery in infectious diseases and as a platform for DNA vaccination [18]. Yet, despite decades of effort focused to enhance drug penetration at the nail level, tattooing has never been investigated as a transungual drug delivery platform. This is notable given its capacity to simultaneously create controlled microchannels and actively infuse liquid formulations into tissue. This approach is precise, reproducible, scalable, and inexpensive, requiring no specialised training and leaving semi-permanent deposits that fade naturally as the nail grows out [19]. Tattooing is generally painless, often producing only a tingling sensation, and the microchannels generated can facilitate both immediate drug infusion and improved penetration during subsequent topical applications, as pores may remain in the nail.

Here we introduce nail tattooing as a disruptive approach to transungual drug delivery. Ciclopirox, a broad-spectrum antifungal, and clobetasol, a potent corticosteroid, were selected as model drugs relevant to onychomycosis and nail psoriasis [20, 21]. We hypothesised that tattooing would accelerate drug flux across the nail by reducing diffusion path length and actively depositing lacquer within microchannels, while leaving conduits to facilitate subsequent topical dosing. To test this, the physicochemical properties of lacquers and tattoo inks were characterised, and drug permeation was quantified using a validated bovine hoof model. Drug distribution and nail microstructure were visualised with confocal microscopy, micro-computed tomography, X-ray diffraction, Raman spectroscopy, and scanning electron microscopy. This work establishes, for the first time, tattooing as a minimally invasive, point-of-care strategy for enhancing drug penetration through the nail. Beyond fungal and psoriatic nail disease, the concept of using tattoo devices as controlled microperforation–infusion systems may open new opportunities in targeted delivery across keratinised barriers.

## Materials and methods

### Materials

Ciclopirox olamine and clobetasol-17-propionate were purchased from Acofarma^®^ (Barcelona, Spain). Tattoo inks were obtained from Stigma (Zhejiang, China). DexULac^®^ and Regenail^®^ were kindly provided by Reig Jofre (Barcelona, Spain). Fluorescein was purchased from Sigma-Aldrich (Missouri, USA). Acetonitrile HPLC LC–MS grade was purchased from VWR Chemicals (Pennsylvania, USA). Orthophosphoric acid was obtained from Merck (Darmstadt, Germany). Ethylenediaminetetraacetic acid (EDTA) was purchased from Labkem (Barcelona, Spain). Hydroxypropyl-β-cyclodextrin (HPβCD) was purchased from Roquette (Lestrem, France).

### Physicochemical characterisation of the formulations

Tattoo inks consist of water-insoluble pigments suspended in a carrier solution containing water, glycerol, binders, surfactants, solvents, and preservatives [17]. DexULac^®^, is a lacquer containing ciclopirox, HPβCD, Pluronic^®^ F127, sodium lauryl sulfate, ethanol, water, and ethyl acetate [22]. Regenail^®^ is a base lacquer with a similar composition but without ciclopirox. For this study, clobetasol was dissolved in Regenail^®^ at 0.3% w/v. In both lacquers, HPβCD enhances drug penetration and Pluronic^®^ F127 prolongs drug retention within the nail. To assess formulation behaviour during tattoo device dispensing, rheological and interfacial properties were evaluated.

#### Density

Aliquots of known volume were dispensed using a micropipette and weighed on an analytical balance (Quintix^®^ 35-1S, Sartorius, Goettingen, Germany). Density (ρ) was calculated as ρ = m/v, where m is mass and v volume. Measurements were performed in triplicate.

#### Viscosity

Viscosity was determined using a rotational viscometer (ViscoQC 300-L, Anton Paar Ltd., Graz, Austria) at 20ºC using a CC12 spindle and a 2 mL sample. The shear rate ranged from 12.91 to 129.1 s^−1^ (10–100 rpm). Tests were performed in triplicate.

#### pH

The pH measurements were conducted using a pH meter (Hanna^®^ HI5522, Hanna Instruments, Bedfordshire, United Kingdom) in triplicate.

#### Surface tension

Surface tension measurements were determined using a microtensiometer (Delta-8, Kibron Inc., Helsinki, Finland). A volume of 50μL of each sample was dispensed into a 96-well plate. Milli-Q^®^ water was used as the calibration solution. Each test was performed in triplicate.

#### Contact angle

Droplets were deposited on nail surfaces and recorded by slow-motion video for 10 s. Contact angles were determined from images captured at droplet stabilisation using ImageJ (National Institutes of Health, Washington, DC, USA). Measurements were performed in triplicate for each sample.

### Nail tattooing process

Fingernail and toenail clipping samples were donated by healthy volunteers (25–65 years) of both sexes after written informed consent. The study protocol was approved by the Santiago-Lugo Research Ethics Committee (Project identification code: 2018/099, Date of approval: 22 February 2018) and was conducted in accordance with the ethical principles of the Declaration of Helsinki (Clinical trial number: not applicable).

**Fig. 1:**
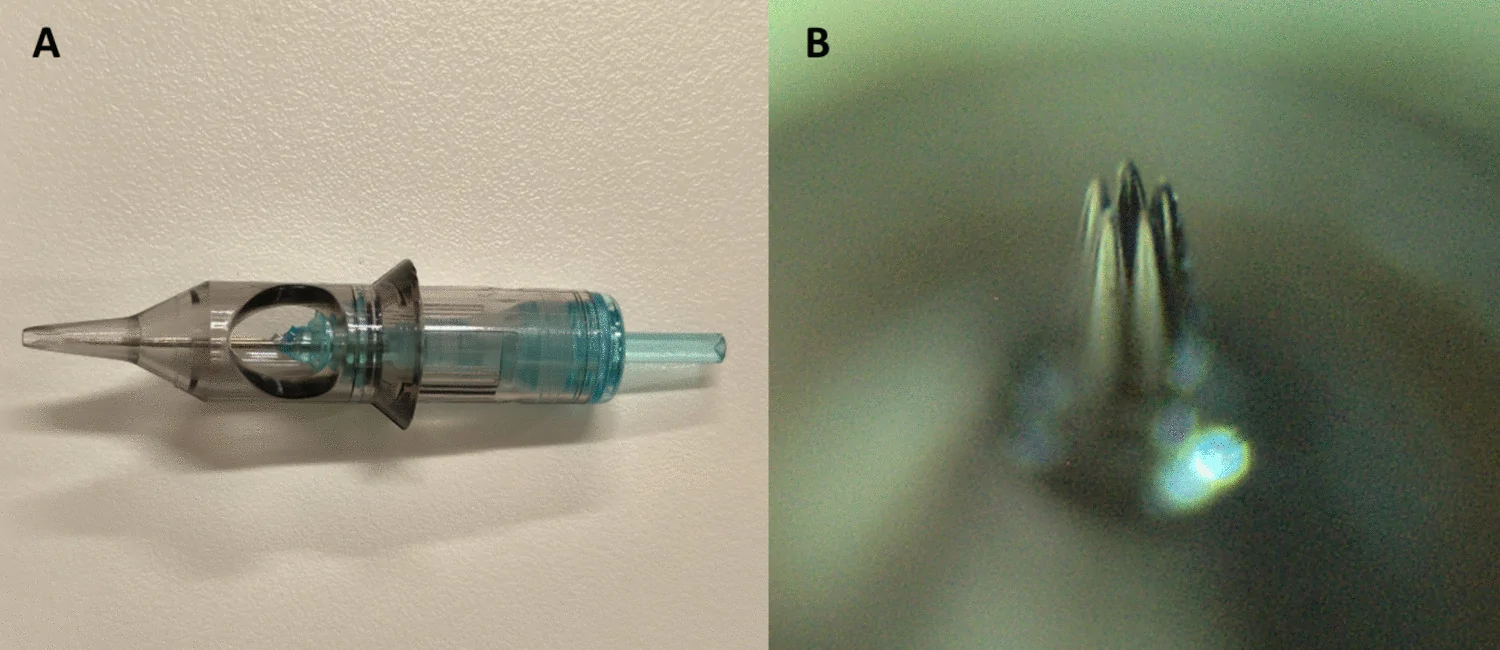
(**A**) Round liner #12 (5RL) sterile disposable needle cartridge. (**B**) Close-up view of the five individual needle tips within the cartridge

Nail tattooing was performed using a Stigma rotary tattoo machine (Zhejiang, China) comprising a battery, a pen-style handpiece, and disposable cartridge needle. Sterile round liner #12 (5RL) cartridges were used. Each needle had a shaft diameter of 0.35 mm, divided into five tips of ~ 50 μm each (Fig. 1). Needle protrusion was set at 1.5 mm. Cartridges were loaded with 0.15 mL of DexULac^®^ or Regenail^®^ with clobetasol. The procedure lasted 30 s, with the needle oscillating at 8,000 rpm (≈4,000 punctures over the nail surface). Gentle pressure ensured penetration into the nail without full perforation. Excess formulation was left in place.

### Drug analytical method

Ciclopirox olamine and clobetasol were quantified by ultra high-performance liquid chromatography (UHPLC) (ACQUITY UPLC H-Class Plus, Waters, Milford, USA) equipped with a FTN injector and PDA detector. Ciclopirox olamine was analysed using an ACQUITY UPLC BEH C18 column (1.7 µm, 2.1 × 50 mm; Waters, USA). The mobile phase consisted of 60% orthophosphoric acid (20 mM) and 40% acetonitrile under isocratic conditions at a flow rate of 0.5 mL/min. The injection volume was 50 µL, the column temperature 30 °C, and detection was performed at 300 nm. The elution time was approximately 0.85 min.

Clobetasol was analysed using an ACQUITY UPLC BEH C18 column (1.7 µm, 2.1 × 50 mm; Waters, USA). The mobile phase consisted of 40% water, 15% acetonitrile, and 45% methanol under isocratic conditions at a flow rate of 0.5 mL/min. The injection volume was 10 µL, the column temperature 40 °C, and detection was performed at 242 nm. The elution time was approximately 1.38 min.

### Permeation studies using a bovine hoof model

Permeation studies employed a bovine hoof membrane model [23], which correlates linearly with human nail permeation [20, 24]. This model was chosen because human nails of sufficient size are difficult to obtain. Bovine hooves were obtained from the local slaughterhouse (Compostelana de Carnes S.L; Santiago de Compostela, Spain), cleaned and hydrated for 24 h before slicing into 0.3–0.7 mm sections with a slicer (Ufesa Professional Slicer FS50, Barcelona, Spain).

Sections were hydrated for 15 min with Milli-Q^®^-water, thickness measured with a digital Vernier calliper, and categorised as non-treated, filed (30 s with cardboard file), or tattooed (Section "Nail tattooing process"). Samples were mounted between polytetrafluoroethylene (PTFE) adaptors (diffusion area 0.196 cm^2^) in Franz diffusion cells (Vidrafoc, Spain; receptor volume 7 mL). Donor compartments received 2 mL of lacquer (DexULac^®^ or Regenail^®^ with clobetasol). Receptor medium was pH 7.4 saline phosphate buffer (PBS) with 0.6 mM EDTA for ciclopirox (to prevent chelation) [25] or pH 7.4 PBS with 5% (w/v) HPβCD for clobetasol. Franz cells were maintained at 37 ± 0.5ºC in dark conditions. At 24 h intervals, 0.5 mL samples were withdrawn and replaced with fresh medium. Drug concentrations were quantified by UHPLC (Section "Drug analytical method"), standardised to 0.196 cm2, and plotted versus time.

After permeation assays, residual drug in hooves was determined. Samples were washed, dried, cut into pieces, weighed, and incubated in 1 mL methanol 5% v/v at 25 °C with agitation for 6 days. Following incubation, 50 μL samples were withdrawn from each vial and diluted in 5 mL Milli-Q^®^ water, filtered through 0.22 μm PES filters, and analysed by UHPLC.

### Laser scanning confocal microscopy

Confocal microscopy analysis was performed with a Leica Stellaris 8 FALCON (Leica Microsystems, Wetzlar, Germany) equipped with the Leica Application Suite X (LAS X) package. To enhance visibility, DexULac^®^ was supplemented with 6.5% (w/v) fluorescein and applied to tattooed, filed, and non-treated nails (n = 3; ~ 3–4 mm each). Tattooed nails were divided into two subgroups: (i) nails tattooed with lacquer-loaded needles and (ii) nails tattooed without lacquer. In the latter case, the tattoo machine was first used to create holes, after which lacquer was applied in the same way as for filed or non-treated nails. This design allowed comparison between direct lacquer infusion during tattooing and post-application onto preformed holes. All nail samples were mounted in vertical Franz diffusion cells (Section "Permeation studies using a bovine hoof model"), with the dorsal surface facing the donor compartment. Samples were excited using a white light laser (WLL2), and FITC was imaged at 491 nm excitation and 496–550 nm emission. mage stacks were acquired at 1024 × 1024-pixel resolution (1550 × 1550 μm XY area, 10 μm Z-steps) using an HC PL APO CS2 10 ×/0.40 dry objective.

### Characterisation of the non-treated, filed, and tattooed nails

#### Stereomicroscopy and scanning electron microscopy (SEM)

The surface morphology of non-treated and tattooed nails (the later treated with a coloured lacquer consisting of Regenail^®^ mixed with red tattoo ink) was examined using a stereomicroscope equipped with an electronic camera (Olympus^®^ SZ-CTV/Olympus^®^ SC100).

For SEM analysis, nail samples (tattooed, filed, and non-treated) were mounted on metal stubs using double-sided conductive adhesive tape, sputter-coated with iridium, and imaged at various magnifications using an analytical scanning electron microscopy (EVO LS 15/EDX, ZEISS, Jena, Germany).

#### X-ray micro computed tomography (Micro-CT)

Nail microstructure was examined with a Skyscan 1272 high-resolution 3D micro-CT system (Bruker, Kontich, Belgium) equipped with an 11 MP detector. Scans were acquired at 5 μm pixel resolution, 50 kV, and 200 μA without filter. Projections were collected over 360º with a 0.4° rotation step and 350 ms exposure time. Images were reconstructed with NRecon and analysed using CTvox software (Bruker).

#### X-ray diffraction analysis (XRD)

X-ray powder diffraction was used to characterise the crystal structure of ciclopirox olamine in DexULac^®^ and in treated/non-treated nails. Analyses were performed in Bragg–Brentano geometry using a Bruker D8 Advance diffractometer (40 kV, 40 mA, θ/θ) with a sealed Cu tube (CuKα1, λ = 1.5406 Å) and LYNXEYE XE-T detector. Diffractograms were collected over a 2θ range of 3–40°, with 0.02° step size and 2 s counting time per step.

#### RAMAN spectroscopy

Raman spectra were recorded at room temperature using an InVia Raman microscope (Renisahw plc, Wotton-under-Edge, United Kingdom) equipped with a 785 nm laser.

Non-treated and filed nail samples were analysed at 1% laser power, 50 × magnification, with acquisition times of 12–13 s and five accumulations. Tattooed nails, tattooed nails with DexULac^®^, and DexULac^®^ formulation were analysed at 5% laser power, 50 × magnification, with 12 s acquisition time and five accumulations.

#### Statistical analysis

All experiments were performed in triplicate unless otherwise stated. Results are presented as mean ± standard deviation. Statistical comparisons between groups (non-treated, filed, and tattooed nails) were conducted using one-way analysis of variance (ANOVA) followed by Tukey’s post hoc test, with significance defined at p < 0.05. Non-significant differences are reported as n.s. Data analysis was performed using GraphPad Prism (version 8, GraphPad Software, USA).

## Results and discussion

### Physicochemical characterisation of the formulations

The development of ink formulations is often empirical. However, optimising properties such as viscosity, density, surface tension, and contact angle is important to achieve optimal tattooing outcomes and prevent issues like needle clogging [26]. The key physicochemical properties of the commercial tattoo inks and the medicated lacquers are summarised in Table 1. The natural pH of the nail plate is approximately 5, and any deviation can indicate structural damage to the nail caused by external products or diseases [27]. While all tested tattoo inks and the Regenail^®^ with clobetasol lacquer exhibited mildly alkaline pH values (above 7), the DexULac^®^ lacquer had a pH of 5.44, closer to the nail’s natural pH. Using a formulation near the nail’s native pH may help prevent damage to the nail’s microstructure during treatment.

**Table 1:** Physicochemical properties of the commercial tattoo inks and nail lacquers

Formulation	pH	Density (g/mL)	Viscosity^*^ (mPa·s)	Surface Tension (mN·m^−1^)	Contact Angle (º)
Red Ink	8.07 ± 0.02	1.08 ± 0.01	33.20 ± 0.30	37.90 ± 6.50	49.75 ± 5.51
Black Ink	7.51 ± 0.05	1.10 ± 0.01	64.03 ± 1.52	49.60 ± 4.50	42.55 ± 7.04
Blue Ink	7.96 ± 0.02	1.10 ± 0.01	67.91 ± 1.64	40.80 ± 1.74	44.56 ± 3.77
Yellow Ink	7.89 ± 0.01	1.07 ± 0.01	111.10 ± 2.95	63.50 ± 5.50	51.43 ± 4.69
DexULac^®^	5.44 ± 0.09	0.92 ± 0.01	8.71 ± 0.08	27.93 ± 0.23	39.21 ± 7.43
Regenail^®^ with clobetasol	7.27 ± 0.02	0.97 ± 0.01	7.20 ± 0.082	26.95 ± 0.18	37.38 ± 7.14

*Viscosity at a shear rate of 103.3 s^−1^

All the commercial tattoo inks exhibited similar densities (1.08—1.10 g/mL). In contrast, DexULac^®^ and Regenail^®^ with clobetasol had lower densities (0.92 and 0.97 g/mL, respectively), which can be attributed to the presence of low-density volatile solvents like ethanol (density 0.789 g/mL) and ethyl acetate (0.900 g/mL) in their formulations.

**Fig. 2:**
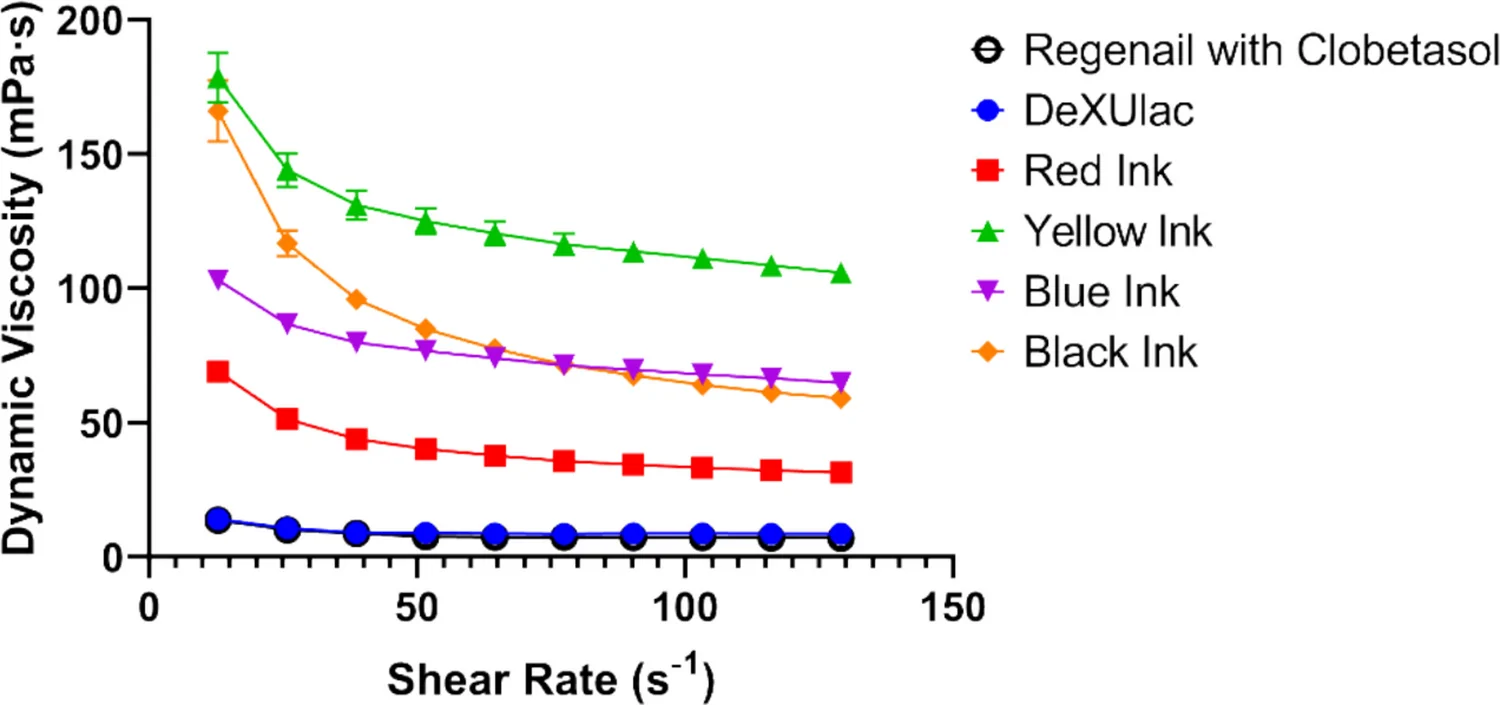
Viscosity as a function of shear rate for the commercial tattoo inks vs. the DexULac^®^ and Regenail.^®^ lacquers (*n* = 3). All formulations show decreasing viscosity with increasing shear (shear-thinning behaviour)

All formulations showed decreasing viscosity with increasing shear rate, indicative of shear-thinning behaviour (Fig. 2). The tattoo inks displayed a wide range of viscosities at the reference shear rate of 103.3 s^−1^: 33.20 ± 0.30 mPa·s (red), 64.03 ± 1.52 mPa·s (black), 67.91 ± 1.64 mPa·s (blue), and 111.10 ± 2.95 mPa·s (yellow). This variability may be attributed to differences in pigment particle concentration and additives in each ink [17].

Notably, viscosity alone does not appear to be a determining factor for the tattooing process, since all these inks are often formulated to be relatively viscous to prevent pigment spreading under the skin (which can cause blurred lines). By comparison, the medicated nail lacquers had much lower viscosities: 8.71 ± 0.08 mPa·s for DexULac^®^ and 7.20 ± 0.082 mPa·s for Regenail^®^ (at a shear rate of 103.3 s^−1^). This low viscosity is appropriate for a lacquer designed to spread over a nail surface. Interestingly, other studies have reported some tattoo inks behaving as Newtonian fluids with viscosities around 0.9–1.2 mPa·s [17], which is an order of magnitude lower than the viscosities observed here for the lacquers. This discrepancy could be due to differences in ink composition or measurement conditions, but it underscores that our lacquer formulations are still relatively fluid compared to certain ink formulations. Importantly, once the tattoo machine withdraws the needle from the nail, capillary action draws the lacquer into the created microchannels [28], so the shear-thinning nature of the lacquer should have minimal adverse effect on its infusion into the needle tracks.

Surface tension and contact angle are also critical parameters for lacquer performance on nails, influencing how well the formulation spreads and adheres. To enhance adhesion to a nail surface, a lacquer should ideally have a surface tension below 30 mN·m^−1^ [29]. Both medicated lacquers in this study exhibited the lowest surface tensions among all the tested formulations (around 27–28 mN·m^−1^, Table 1) and relatively low contact angles on the nail surface (~ 37–39°). Despite their lower surface tension (compared to the tattoo inks), we observed that neither lacquer dripped from the needle during tattoo machine operation; the formulations were released only upon contact with the nail. The inclusion of surfactants such as sodium lauryl sulfate and Pluronic^®^ F127 in the lacquers likely contributes to the reduced surface tension and contact angle [30]. These properties improve the wetting and spreading of the lacquer on the nail plate and, in conjunction with the microchannels created by the tattooing process, may facilitate deeper penetration of DexULac^®^ and Regenail^®^ into the nail compared to the more surface-active tattoo inks.

### Nail tattooing procedure and nail preparation

The nail tattooing was performed using a single-needle tattoo cartridge (one-pin head), which was chosen over multi-needle configurations (e.g., five-pin heads) to maximize control and precision. Rigorous preliminary testing was conducted to ensure that the needle would not fully pierce through the nail plate under various settings. It was confirmed that, under the selected conditions, the needle created channels in the nail without passing completely through it. An intermediate tattoo machine operating frequency and a moderate needle protrusion were found to offer the most comfortable and controlled conditions for the operator, while still reliably perforating the nail surface.

The tattoo machine was calibrated so that the needle tip extended 1.5 mm beyond the handpiece’s barrel guide. During tattooing, the operator applied gentle pressure, just enough to allow the needle to punch into the nail’s upper layers without reaching the nail bed. Each nail was tattooed for approximately 30 s, during which the loaded formulation (ink or lacquer) was completely delivered into the nail. Any excess formulation on the nail surface was intentionally left in place (not wiped off) to allow continued absorption. To maintain hygiene and prevent cross-sample contamination, a new sterile needle cartridge was used for each nail and immediately discarded after use.

Although the manufacturers of DexULac^®^ and Regenail^®^ do not require nail filing before applying these lacquers, a pre-filing condition was included in the present study for comparison. In the “filed” group, each nail was lightly filed over its entire surface (ensuring the file made at least one pass over every area of the nail plate) prior to any treatment. This filing aimed to remove superficial gloss and create shallow grooves, which is known to increase lacquer adhesion and penetration by reducing the physical barrier of the dense nail surface. The outcomes on filed nails offer a benchmark for how traditional mechanical preparation compares to the novel tattooing approach.

### Permeation studies using a bovine hoof model

**Fig. 3:**
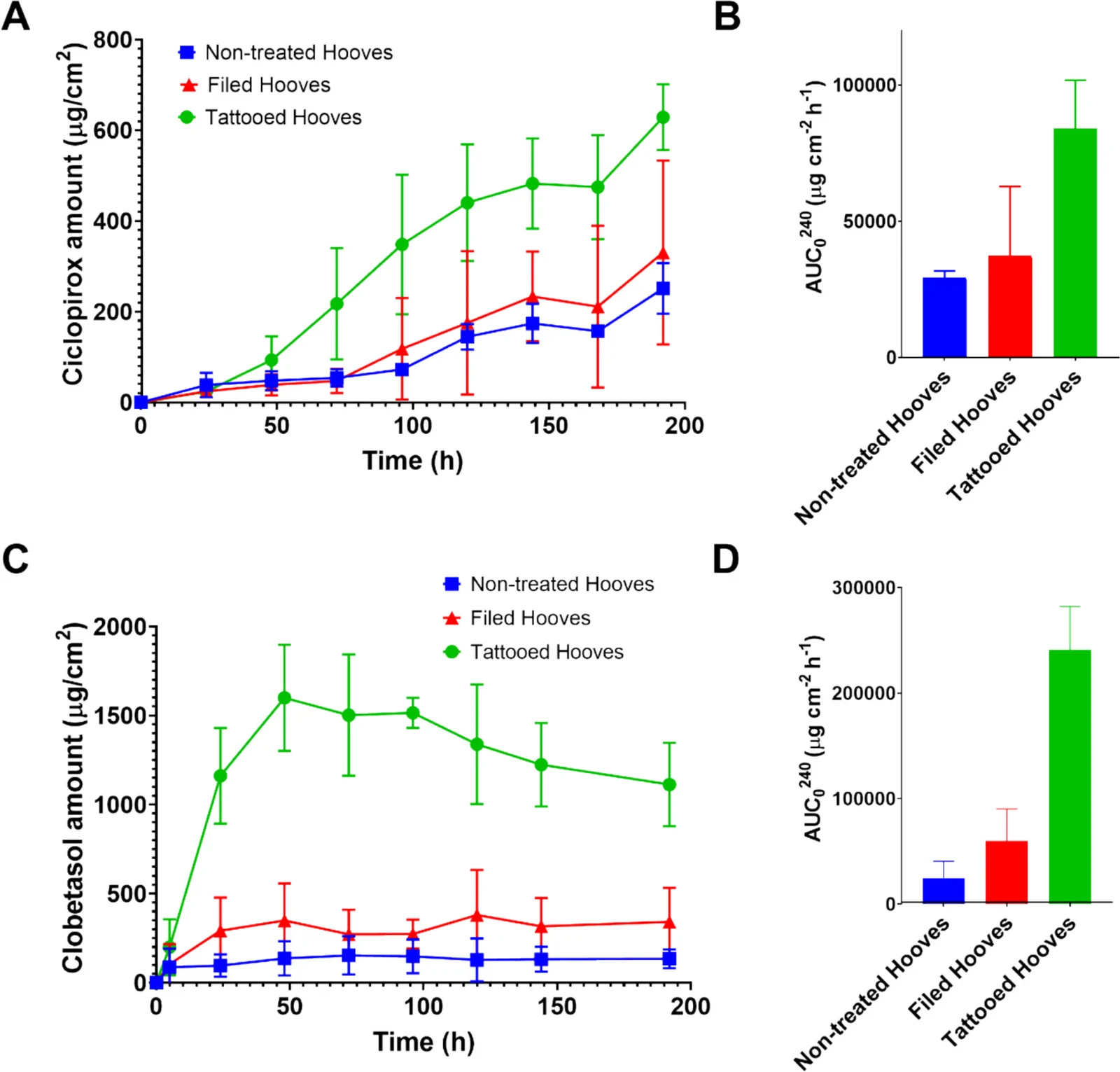
(**A**) Cumulative ciclopirox permeation through non-treated, filed, and tattooed bovine hooves over 8 days (*n* = 3). (**B**) AUC_0_^t^ for ciclopirox permeation in each group, illustrating total drug delivered (tattooed vs. filed vs. untreated). (**C**) Cumulative clobetasol permeation profiles for the three conditions (*n* = 3). (**D**) AUC_0_.^t^ for clobetasol permeation in each group. Tattooed hooves show significantly enhanced permeation and AUC for both drugs compared to controls (α < 0.05)

Figure 3A and C show the 8-day permeation profiles of ciclopirox olamine and clobetasol, respectively, through non-treated, filed, and tattooed bovine hooves. In the case of ciclopirox (Fig. 3A), the cumulative permeation curve initially rose in a curvilinear fashion (characteristic of a non-steady state diffusion period) and later progressed to a roughly linear increase (indicating steady-state diffusion). Notably, the hoof samples that underwent tattooing showed a markedly higher ciclopirox penetration rate. By day 8, the tattooed hooves had a significantly greater amount of ciclopirox permeated compared to both the non-treated and filed hooves (α < 0.05). This enhanced delivery is attributed to the structural modifications introduced by tattooing, as the network of microchannels (holes) created by the needle increases the contact area between the lacquer and the hoof and provides direct pathways for the drug to diffuse deeper into the nail plate.

The steady-state portion of the ciclopirox permeation curve can be further analysed using Fick’s first law of diffusion. According to this model, the drug flux at steady state (J) is proportional to the permeability coefficient (P) of the drug in the nail and the concentration gradient across the nail. The permeability coefficient can be expressed as:

1 $$P=D\bullet \frac{K}{L}$$

where D is the diffusion coefficient of the drug in the nail plate, K is the drug’s partition coefficient between the lacquer and nail, and L is the thickness of the nail plate.

The tattoo-induced holes effectively reduce the diffusion path length L (since the drug only needs to traverse from the bottom of a microchannel into the lower nail layers) and simultaneously increase the surface area available for drug uptake. According to Eq. 1, both changes would increase the apparent permeability P. In practical terms, the tattooing process shortens the distance the drug must travel to reach the deeper regions of the nail plate and provides more entry points, thus facilitating faster drug penetration into the hoof’s internal layers compared to the filed or non-treated hooves.

Clobetasol exhibited a similar improvement in permeation with the tattooing method. As shown in Fig. 3C, tattooed hooves achieved higher clobetasol permeation over 8 days than either filed or non-treated hooves (p < 0.05). This indicates that the benefit of tattoo-created channels is not limited to one type of drug or formulation but can enhance the delivery of both a water-soluble antifungal agent (ciclopirox olamine) and a corticosteroid (clobetasol) with different physicochemical properties.

Apparent permeability (cm/s), flux (µg/cm^2^·h), and lag time (h) data were calculated according to Fick’s first law and are presented in Table 2. Calculated permeation parameters differed across hoof treatments. For ciclopirox, were 2.00 µg/cm^2^·h (non-treated), 1.28 µg/cm^2^·h (filed) and 4.88 µg/cm^2^·h (tattooed hooves). For clobetasol, corresponding values were 14.02, 6.89 and 31.33 µg/cm^2^·h, respectively. In both cases, tattooed hooves exhibited the highest permeation rates. This enhancement is likely attributable to the creation of microchannel that decrease the structural resistance of the keratinized matrix. In transungual delivery, drug transport is government by the interplay between diffusivity (D), partition coefficient (K) and barrier thickness (h), and physical disruption of the nail plate has been shown to significantly alter these parameters.

**Table 2:** Permeation parameters calculated according to Fick’s first law for ciclopirox and clobetasol in bovine hoof

Ciclopirox			
Application	Flux (µg/cm^2^·h)	Apparent Permeability (cm/s)	Time Lag (h)
Non-treated Hooves	2.00	7.38·10^–7^	36.68
Filed Hooves	1.28	4.73·10^–7^	21.82
Tatooed Hooves	4.88	1.79·10^–6^	27.64
Clobetasol			
Application	Flux (µg/cm^2^·h)	Apparent Permeability (cm/s)	Time Lag (h)
Non-treated Hooves	14.02	6.49·10^–4^	-
Filed Hooves	6.89	3.23·10^–4^	-
Tatooed Hooves	31.33	1.45·10^–3^	-

Apparent permeability coefficients followed the same trend as flux values. For ciclopirox apparent permeability was 1.79·10⁻⁶ cm/s in tattooed hooves, compared with 4.73·10⁻⁷ and 7.38·10⁻⁷ cm/s in non-treated hooves and filed hooves, respectively. For clobetasol, the value obtained for tattooed hooves was 1.45·10–3 cm/s, while for untreated and filed hooves it was 3.23·10–4 cm/s and 6.49·10–4 cm/s, respectively. The higher permeation observed for clobetasol relative to ciclopirox is consistent with its greater lipophilicity, which may enhance its affinity for the keratin matrix [31].

Lag time was determined only for ciclopirox, as clobetasol was detected in the first sampling interval, precluding reliable estimation of lag time between the beginning of the assay and the collects of first sample. The shorter apparent latency for clobetasol suggests faster establishment of steady-sate diffusion, which may reflect differences in molecular affinity and diffusional behavior within the keratinized substrate.

**Fig. 4:**
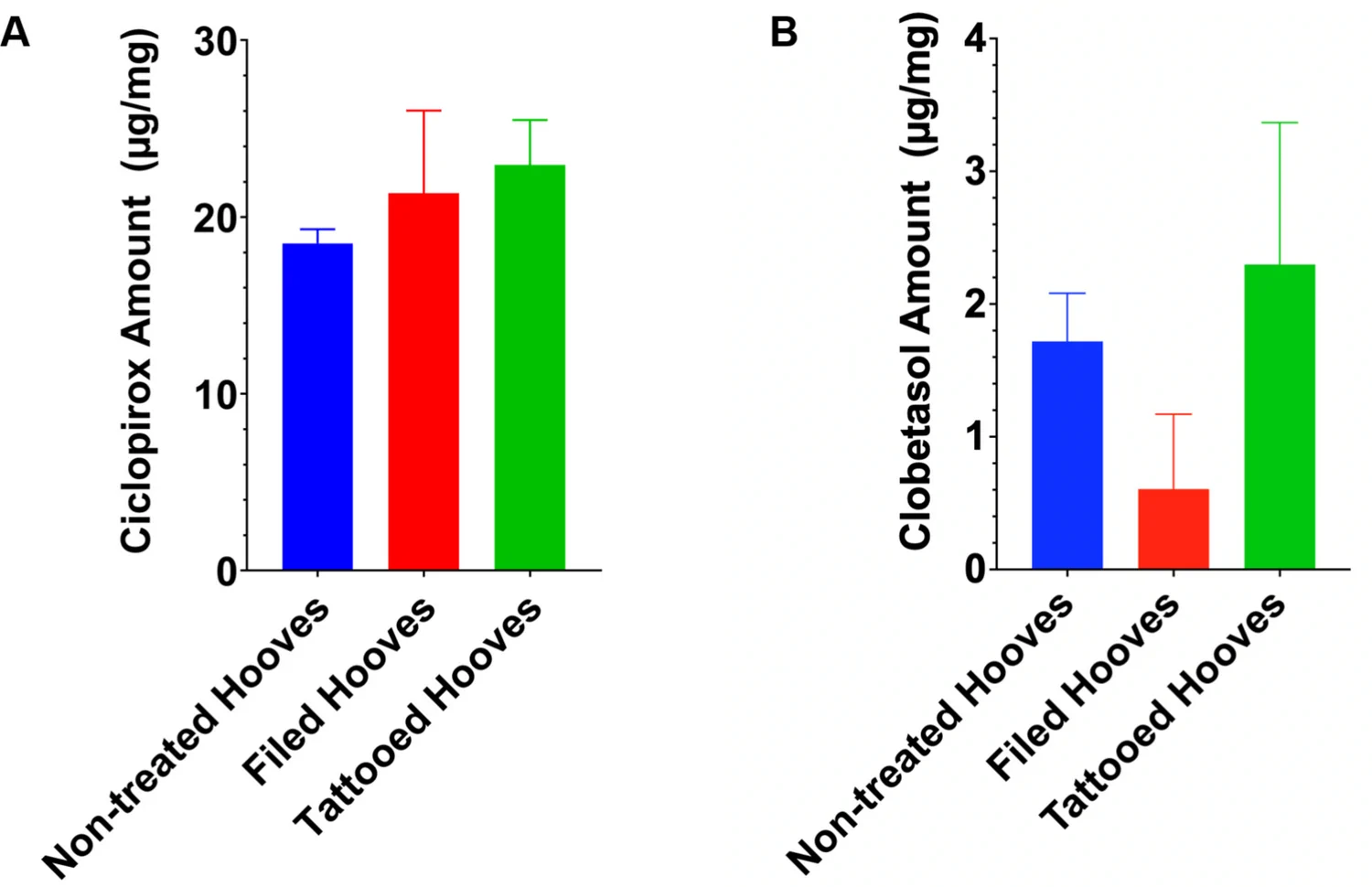
Amount of (**A**) ciclopirox and (**B**) clobetasol extracted from the hoof (per gram of hoof tissue) after the 8-day permeation study, for non-treated, filed, and tattooed conditions. Despite faster and higher permeation in tattooed hooves, the total drug accumulated in the hoof by the end of the study was similar across groups for each drug (α = n.s. for ciclopirox; clobetasol showed a similar pattern), indicating that tattooing primarily accelerates drug transport without changing the nail’s ultimate drug capacity

Values of apparent permeability and flux must be interpreted with caution. The tattooing procedure modifies the physical integrity and potentially the effective permeation area of the hoof sample. Since flux and apparent calculations are normalized to the projected surface area, any increase in true diffusional area or alteration in microstructure may lead to overestimation of intrinsic permeability parameters. Figure 3B and D present the area under the permeation curve from time 0 to the final time t (AUC_0_^t^) for ciclopirox and clobetasol, respectively, across the three treatment groups. The AUC_0_^t^ can be a useful metric derived from the permeation profile (cumulative amount permeated vs time) (Fig. 4A and C), providing an integrative measure of drug transport into the receptor compartment of the Franz diffusion cells over the 8-day period, rather than just single time-point readings. Similar integrative descriptor derived from permeation profiles have been used in in vitro permeation studies, where the cumulative amount permeated can be related to the AUC of the permeation profile [32]. Consistent with the individual time-point data, the tattooed hooves showed the highest AUC values for both ciclopirox and clobetasol. Quantitatively, the total permeation AUC_0_^t^ achieved with tattooing was significantly greater than that of the non-treated and filed hooves for both drugs (p < 0.05). This reinforces that tattoo pretreatment provides a superior cumulative delivery of medication into the nail over time.

After completing the 8-day permeation studies, the hoof samples were thoroughly cleaned to remove any lacquer residue from the surface. Then, the ciclopirox and clobetasol accumulated within the hoof tissue (nail plate) itself were extracted and quantified. The results (Fig. 4) are expressed as drug amount per unit hoof mass for each treatment group. Interestingly, for ciclopirox (Fig. 4A), there were no significant differences in the total drug accumulation in the hoof among the non-treated, filed, and tattooed groups by the end of the experiment (p > 0.05, i.e. not statistically significant). In other words, although tattooing greatly accelerated the rate of ciclopirox penetration (as seen in the higher flux and AUC), by day 8 the overall amount of ciclopirox retained in the nail plate was comparable across all conditions. This outcome suggests that the nail may reach a saturation or equilibrium concentration of ciclopirox that is dictated by the drug’s partitioning and solubility in the nail matrix, regardless of the delivery method. The tattooing process primarily speeds up how fast that equilibrium is achieved (higher flux), without necessarily increasing the equilibrium concentration itself. Clobetasol showed a qualitatively similar trend, as the tattooed hooves had much faster initial drug uptake, but the final accumulated amount in the nail by day 8 was not dramatically higher than in the other groups (Fig. 4B).

Although tattooing did not consistently increase total drug accumulation within the nail plate, it significantly enhanced drug permeation across the nail. In onychomycosis, the primary site of infection is located in the nail bed and hyponychium rather than within the nail plate itself. Therefore, successful treatment depends on delivering therapeutically relevant drug concentrations across the nail barrier to reach the underlying infected tissue. Increased transungual permeation, reflected by higher cumulative permeation and reduced diffusion barriers, is therefore more relevant to therapeutic efficacy than drug retention within the nail plate alone. The microchannels created by tattooing reduce the effective diffusion path length and facilitate direct transport toward the nail bed, potentially improving drug delivery to the site of infection, particularly in moderate to severe disease where passive diffusion from conventional lacquers is insufficient.

### Laser scanning confocal microscopy

**Fig. 5:**
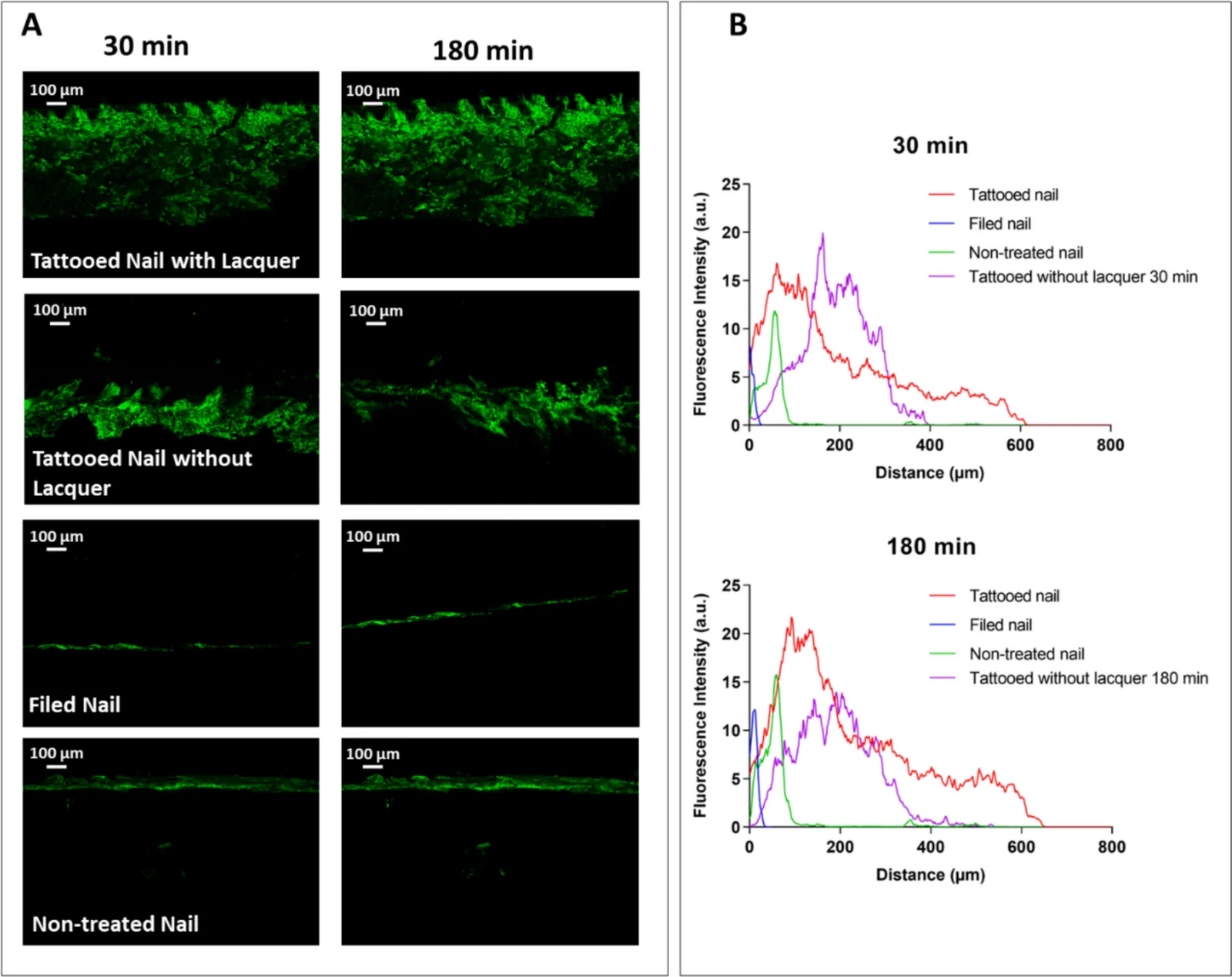
(**A**) Cross-sectional laser scanning confocal microscopy images showing the distribution of DexULac.^®^ (green fluorescence) in non-treated, filed, and tattooed nails. Images are shown at 30 min and 180 min after lacquer application. The tattoo-created channels are visible in the nail cross-sections and appear filled with the fluorescent lacquer immediately upon application. (**B**) Quantitative fluorescence intensity profiles (green signal) as a function of depth from the nail surface, for the different conditions at 30 min and 180 min. Tattooed samples show significantly deeper penetration of the lacquer (hundreds of microns) compared to non-treated or filed nails (tens of microns)

To visualise how the lacquer penetrates and distributes within the nail, laser scanning confocal microscopy on cross-sections of non-treated, filed and tattooed nails after applying DexULac^®^ was performed. The DexULac^®^ lacquer contains a fluorescent component (appearing green in the images), which allows to track its location over time at various depths. Figure 5A presents representative cross-sectional confocal images, and Fig. 5B shows the quantitative fluorescence intensity profiles as a function of depth for each condition.

In non-treated and filed nails (no tattoo pretreatment), the confocal images revealed a bright green band of fluorescence localised at the nail surface, indicating that most of the lacquer remains on top or within the very superficial layers. In these cases, the highest fluorescence intensity was observed at the surface even after several hours, with the signal decaying rapidly with depth. Specifically, in the filed nails, DexULac^®^ could be detected only down to roughly ~ 30 μm below the surface, whereas in non-treated (unfiled) nails the lacquer penetrated slightly deeper, up to ~ 100 μm (Fig. 5B, profiles). This behaviour may be explained by structural alterations induced by filing, including compaction of the superficial keratin layers and partial obstruction of microporosities by keratin debris. Over the 3 h observation period, both non-treated and filed nails showed only a modest increase in fluorescence in the deeper layers, reflecting the slow and limited diffusion of the lacquer into intact or even filed nail plates.

Tattoo pretreatment, in contrast, enabled much deeper and more uniform penetration of the lacquer. Two tattoo-assisted scenarios were assessed: (a) “tattooed nails with lacquer,” meaning the lacquer was loaded in the tattoo needle and injected into the nail during tattooing, and (b) “tattooed nails without lacquer,” meaning the nail was tattoo-perforated with an empty needle (creating microchannels) and then the lacquer was applied onto the surface with a brush. Both approaches resulted in dramatically enhanced lacquer distribution compared to non-tattooed nails. When the nail was first perforated and then lacquer was topically applied (condition b), fluorescence extended from the surface to ~ 450 μm into the nail. In contrast, when the lacquer was injected directly through the tattoo machine (condition a), penetration reached ~ 600–650 μm, reflecting the additional pressure-driven infusion of the formulation into the nail (Fig. 5B). These depths are an order of magnitude greater than what was observed in the non-tattooed nails, demonstrating the profound effect of the tattooing technique on delivery.

The confocal images in Fig. 5A clearly show the physical microchannels created by the tattoo needle, visible as vertical fluorescent pores filled with lacquer. In both tattoo-assisted conditions, an intense fluorescence was present not only at the surface but also emanating from within these channels immediately after lacquer application, confirming that the pores were effectively loaded with the formulation. The difference in penetration depth between the two tattoo conditions (injected vs. post-filled) reflects the mode of lacquer delivery. When the lacquer is injected under pressure though the tattoo needle, the force of the needle perforation actively drives it deeper, reaching closer to the channel bottoms. By contrast, when lacquer is applied onto pre-formed channels, it enters more gradually and tends to remain confined to the upper and mid-sections of the channels. Despite this difference, both tattoo-assisted methods achieved far deeper penetration than could be achieved by surface application alone. These results highlight that creating mechanical pathways in the nail (through tattooing) significantly alters the distribution profile of a topical antifungal lacquer, allowing it to reach deeper resident fungal colonies that would otherwise be inaccessible.

### Characterisation of nail microstructure

The structural characterisation performed in this study serves two complementary purposes: (i) to confirm that the tattooing procedure generates controlled and reproducible microchannels within the nail plate, and (ii) to assess whether this intervention compromises the intrinsic keratin architecture of the nail. Both aspects are critical when evaluating the translational potential of this technique.

**Fig. 6:**
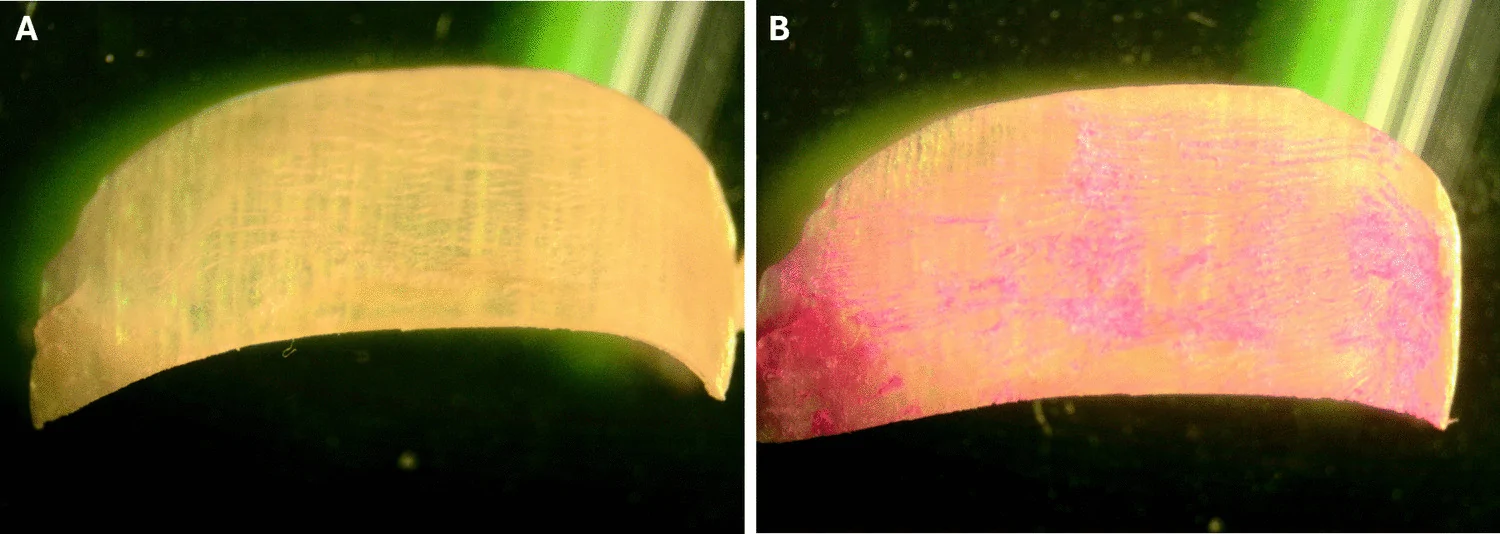
Stereomicroscopy images of nail plates under different pretreatments: (**A**) non-treated (intact) nail showing a smooth and uniform structure, and (**B**) tattooed nail with coloured lacquer (Regenail^®^ mixed with red tattoo ink) showing no visible micropores

Stereomicroscopy revealed no appreciable differences between non-treated (Fig. 6A) and tattooed nails (Fig. 6B). A faint red hue was visible in tattooed samples due to the colored lacquer (Regenail^®^ mixed with red tattoo ink), but no structural disruptions such as micropores were apparent. Overall, the surface remained unchanged intact, indicating that tattooing does not alter the macroscopic appearance of the nail.

**Fig. 7:**
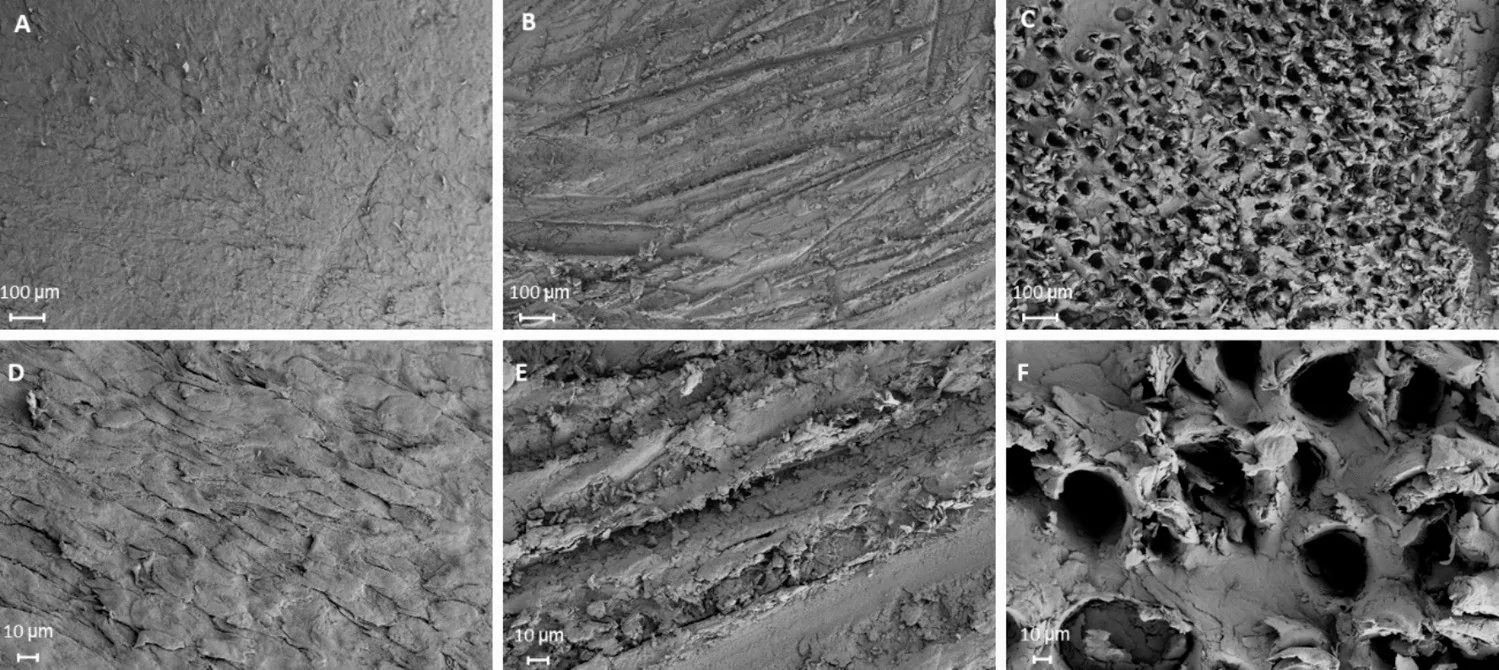
Top-view SEM images of nail plates under different pretreatments: (**A**, **D**) non-treated (intact) nail showing smooth, cohesive keratinised cells; (**B**, **E**) filed nail showing abrasive grooves, removed keratin layers, and micro-cracks; (**C**, **F**) tattooed nail (no lacquer applied) showing numerous uniform micro-holes (~ 50 µm diameter) created by the tattoo needle. Panels **D**, **E**, **F** are higher-magnification views of **A**, **B**, **C** respectively, highlighting surface details

In contrast, SEM analysis revealed distinct surface morphologies. Non-treated nails (Fig. 7A, D) displayed a compact, cohesive arrangement of flattened keratinised cells with minimal surface irregularities, consistent with the known dense dorsal layer morphology. Filed nails (Fig. 7B, E) showed parallel grooves, microdamage, and loss of keratinised structures, reflecting mechanical abrasion and partial removal of keratin layers. Tattooed nails without lacquer (Fig. 7C, F) exhibited discrete circular perforations of ~ 50 μm in diameter, consistent with the needle gauge used. Importantly, these perforations were well-defined and spatially separated rather than diffuse or irregular disruptions.

**Fig. 8:**
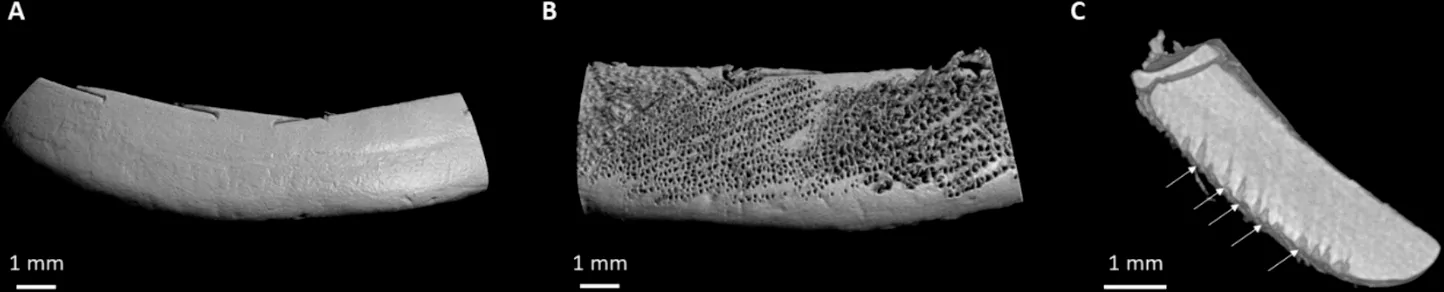
Micro-CT reconstructions of nails: (**A**) untreated nail (surface view) with no holes; (**B**) tattooed nail (surface view) showing the array of micro-channels created by the tattoo machine; (**C**) cross-sectional view of a tattooed nail, illustrating the depth of penetration of the channels (approximately 0.26 mm deep) relative to the total nail thickness (1.18 mm). The tattooing creates controlled-depth channels without fully penetrating the nail plate

Micro-CT imaging provided three-dimensional insights into the nail architecture and the perforations created by tattooing (Fig. 8). In tattooed nails without lacquer, vertical channels extended 257.5 ± 42.5 μm into a plate 1.18 ± 0.14 mm thick, without breaching the full thickness. Untreated nails showed no disruptions. This finding is clinically relevant, as it demonstrates that the procedure creates controlled-depth microconduits while avoiding penetration into the nail bed. Preservation of the ventral plate and subungual tissue is essential to minimise pain, bleeding, and infection risk. The ability to reproducibly limit channel depth supports the safety profile of the technique and distinguishes it from more aggressive mechanical interventions.

**Fig. 9:**
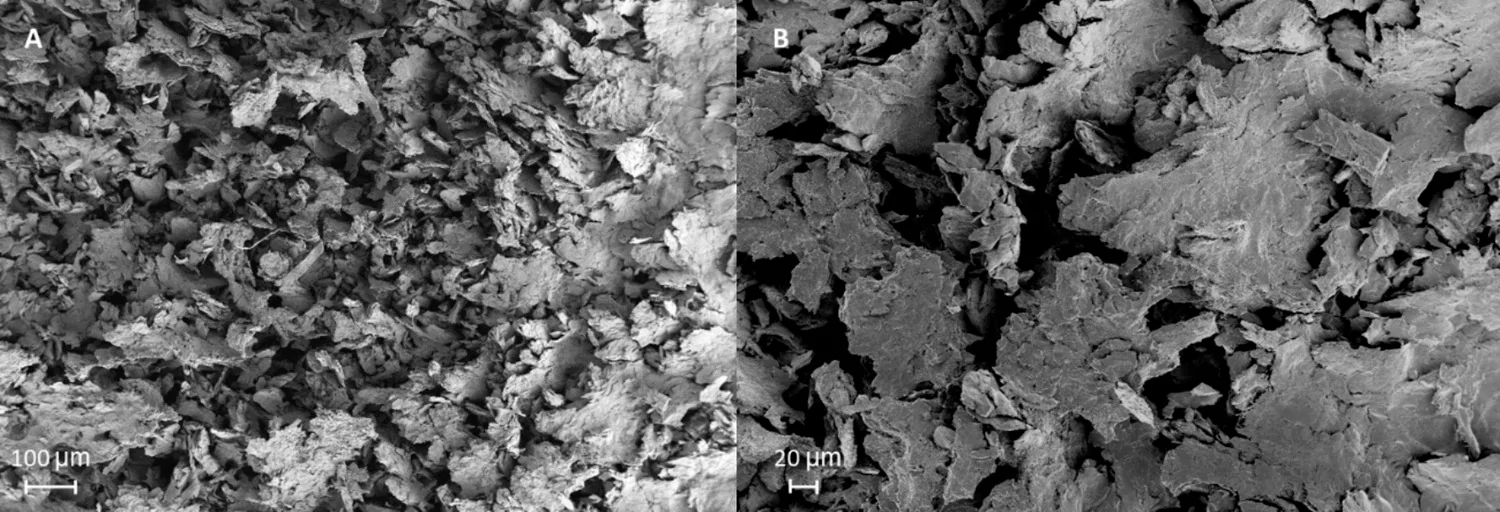
SEM images of a tattooed nail after applying DexULac^®^ lacquer. The surface is uniformly covered by dried lacquer residues (crystalline structures), and the micro-holes are filled/coated by the formulation, making them indistinguishable on the surface

After tattooing with DexULac^®^, SEM no longer distinguished individual openings (Fig. 9). Volatile solvents (ethanol, ethyl acetate) evaporated rapidly, and residual excipients formed crystalline/film-like deposits that covered the surface and visibly filled or capped the channels.

**Fig. 10:**
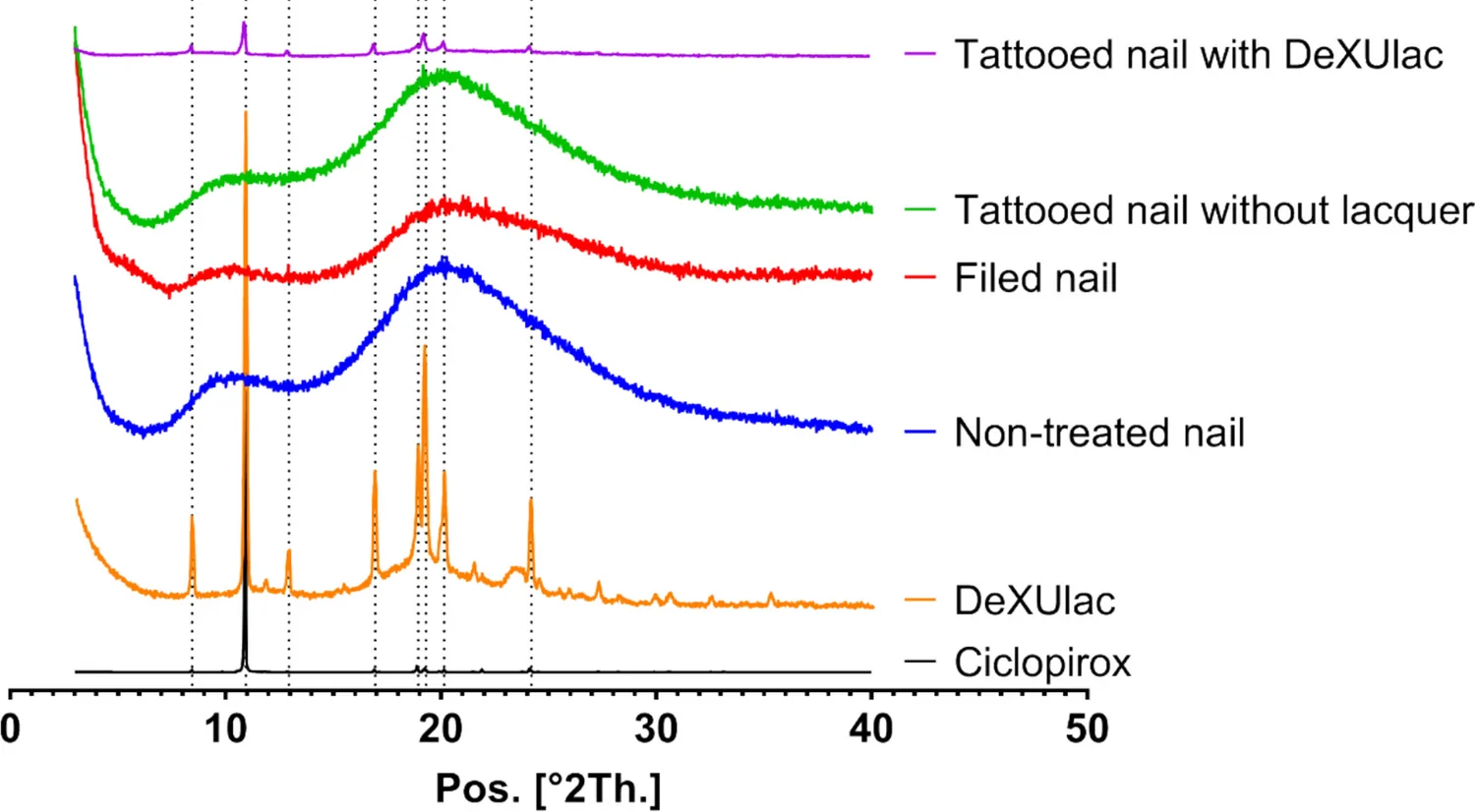
X-ray diffractograms for pure ciclopirox powder, DexULac^®^ lacquer, non-treated nail, filed nail, tattooed nail (no lacquer), and tattooed nail after DexULac^®^ application

X-ray diffraction (Fig. 10) supported these observations. The DexULac^®^ film presented noticeable peaks at 2θ = 8.45, 10.95, 12.95, 16.95, 18.95, 19.30, 20.15, and 24.20, attributable mainly to crystalline ciclopirox. In contrast, non-treated, filed, and tattooed nails (no lacquer) exhibited the characteristic α-keratin peaks at 2θ = 9 and 20 [33]. The reflection at 9° corresponds to the spacing between α-helical axes, while the peak at 20° reflects the periodic distance of the 3.6 residues per turn in the α-helix structure of keratin. In filed nails, these characteristic α-keratin peaks appeared weaker and slightly shifted to lower angles, consistent with alterations in crystalline domain organisation and partial disruption of α-helical order caused by mechanical abrasion. By contrast, tattooed nails without lacquer exhibited diffraction profiles nearly identical to untreated nails, indicating that the tattooing process itself did not measurably affect the crystallinity of α-keratin. In tattooed nails treated with DexULac^®^, the XRD pattern differed markedly from untreated, filed, and tattooed controls. The characteristic α-keratin reflections at 9° and 20° were no longer visible. Instead, sharp peaks corresponding to crystalline ciclopirox dominated the diffractogram, confirming deposition of the drug within the nail in its crystalline state.

**Fig. 11:**
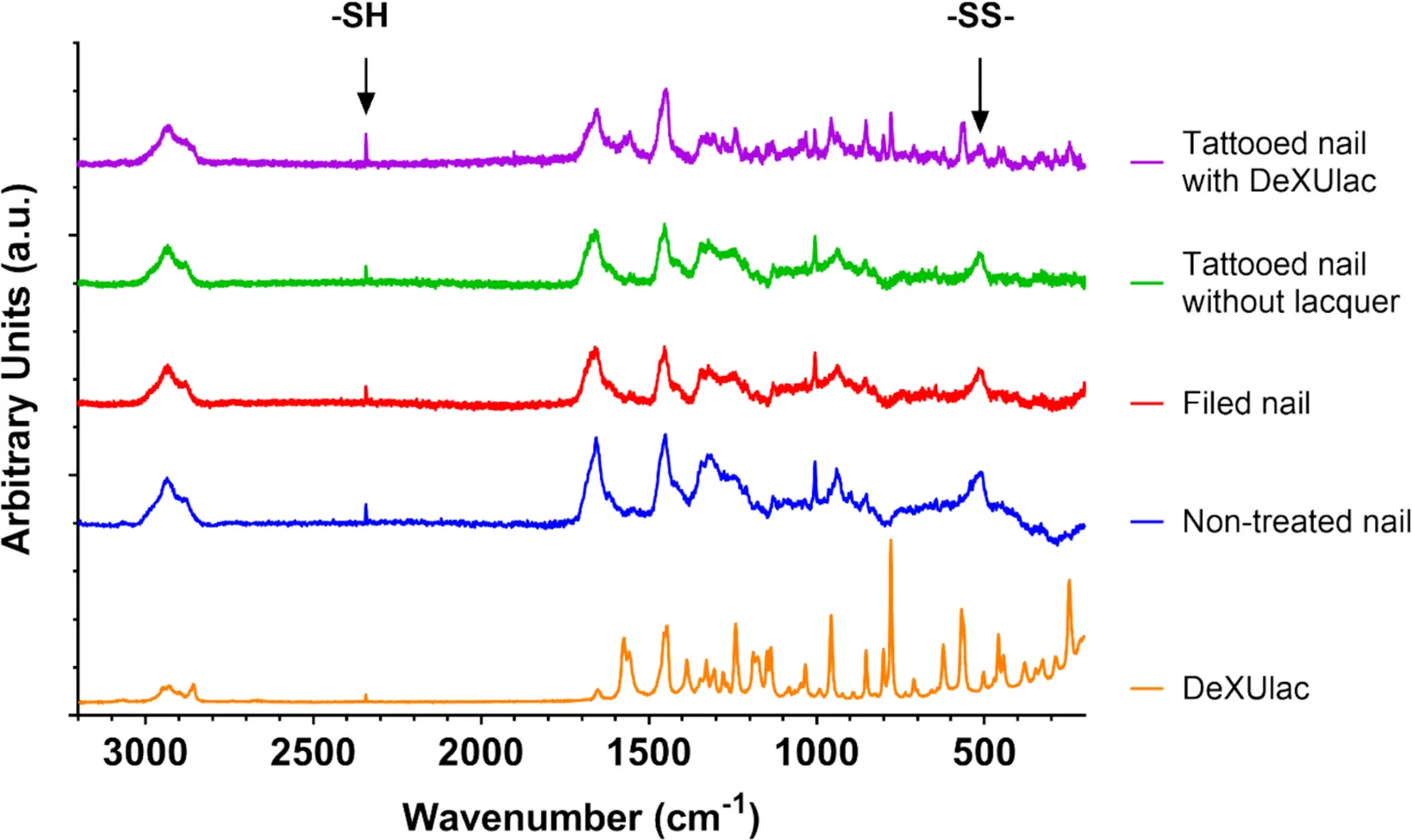
Raman spectra from the surface of DexULac^®^ lacquer film, non-treated nail, filed nail, tattooed nail (no lacquer), and tattooed nail after DexULac^®^ treatment

The Raman spectra in Fig. 11 include the dorsal layer of DexULac^®^, along with untreated, filed, tattooed (without lacquer), and tattooed (with DexULac^®^) nails. The disulfide bond (S–S) of keratin, essential for nail microstructure, is consistently observed as a band at 430–550 cm⁻1 [34]. Cleaved disulfide bonds generate thiol (S–H) groups, which appear as a band at 2550–2600 cm⁻1 [35]. Across untreated, filed, and tattooed nails, the relative intensities of the S–S and S–H bands did not differ significantly (α n.s.), indicating that neither filing nor tattooing disrupted the keratin disulfide network. This is a particularly important observation, as the integrity of disulfide crosslinks underpins the mechanical stability and barrier function of the nail plate. In contrast to chemical penetration enhancers, which may cleave disulfide bonds and weaken the keratin matrix, the tattooing approach enhances delivery through physical microchannel formation without altering keratin chemistry.

The spectra obtained from tattoo-created holes after DexULac^®^ application displayed additional bands corresponding to lacquer components, confirming that the device effectively deposits the formulation within the perforations while preserving the underlying keratin structure.

Taken together, SEM and micro-CT confirmed that tattooing generated uniform pores (~ 50 μm diameter, ~ 250 μm depth) without penetrating the full nail plate. Structural analyses demonstrated that these controlled microchannels preserved the integrity of nail keratin while enabling efficient drug deposition. Non-treated and tattooed nails showed the characteristic α-keratin reflections and intact disulfide bonds, whereas filed nails exhibited partial structural disruption. In tattooed nails treated with DexULac^®^, keratin peaks were replaced by crystalline ciclopirox reflections, and additional Raman bands corresponding to lacquer components were detected, verifying that the formulation was deposited within the nail without compromising keratin integrity.

The macroscopic relevance of these findings lies in the balance between permeability enhancement and structural preservation. Effective transungual drug delivery strategies must overcome the diffusion barrier posed by densely cross-linked keratin, yet excessive structural damage can compromise nail strength, increase brittleness, and potentially predispose to secondary infection. The present results demonstrate that tattooing achieves enhanced permeability primarily by reducing effective diffusion path length and increasing surface area via discrete microchannels, rather than by degrading the keratin network itself.

The nail tattooing technique presented in this work offers a straightforward and practical solution for the treatment of onychomycosis, eliminating the need for complex drug delivery strategies and multi-step treatment regimes. It also avoids the side effects associated with oral therapies. Previous approaches such as nanoparticles [36], UV curable gels [37], and nail microneedles and patches [38], have shown partial success but face significant translational challenges in reaching clinical applications.

This technique holds promise for improving onychomycosis treatment in cases where infection resides in deeper nail layers, such as proximal subungual onychosis. In a clinical setting, specialised tattoo machines designed for this purpose could be employed by podiatrists or physicians. During an initial treatment session, patients would receive nail lacquer application through the tattoo device, using disposable needles to target the affected nail. Subsequently, patients would follow the prescribed treatment regimen outlined in the lacquer’s technical sheet. The punctures created by the tattoo machine would persist in the nail, facilitating enhanced penetration of the lacquer during subsequent applications. In follow-up appointments, clinicians could repeat the tattoo procedure if necessary to sustain therapeutic efficacy. This approach ensures targeted and potentially more effective delivery of the antifungal lacquer to infected areas of the nail. Its application could also be extended to include drug combinations for improved antifungal activity. Tattoo machines are generally regarded as safe products, primarily used for cosmetic purposes, and are required to comply with the provisions of the General Product Safety Directive 2001/95/EC [39]. However, ongoing discussions in the European Parliament are considering their reclassification as medical devices [40]. In addition, the FDA Modernization of Cosmetics Regulation Act (MoCRA) of 2022 requires new tattoo machines to meet medical-grade standards [41], potentially accelerating their integration into clinical practice.

The nail tattooing technique effectively addresses a major hurdle faced by topical therapy: achieving enhanced penetration through the nail plate to treat underlying infection [16]. Limited bioavailability, a key cause of reduced efficacy in topical treatments, has traditionally been addressed with permeation enhancers. Alternative device-based strategies, such as iontophoresis, ultrasound, and laser poration, have shown promising results and are increasingly used in clinical practice [42]. Despite being invasive, the minimal discomfort reported by users of nail tattooing machines may provide an advantage over these alternatives. Moreover, the simplicity of application, absence of bulky or expensive equipment, and the creation of permanent structural changes in the nail make tattooing an appealing option, while allowing patients to continue treatment at home. This eliminates the need for new formulations specifically designed for nail delivery. Furthermore, because lacquer is directly injected into the nail, physicochemical constraints that limit other approaches become less relevant. Given these advantages, future work should explore the application of a wider range of antifungal agents and further evaluation with established ex vivo and in vitro systems could be used to compare tattoo-assisted delivery with conventional topical application under controlled conditions. Such studies would help determine whether the enhanced transungual permeation observed translates into improved drug delivery to the site of infection, paving the way for the first clinical studies of nail tattooing in human onychomycosis. Antifungal agents or their combinations can be easily applied to the nail, using already marketed lacquers such as DexULac^®^, as demonstrated in this study. This eliminates the need for new formulations specifically designed for nail delivery. Moreover, because lacquer is directly injected into the nail, physicochemical constraints that limit other approaches become less relevant. Given these advantages, future work should explore the application of a wider range of antifungal agents, paving the way for the first clinical studies of nail tattooing in human onychomycosis.

## Conclusion

In this study, the nail tattooing was applied for the first time as a strategy to treat fungal nail infections. Nail lacquers containing ciclopirox, used in the treatment of onychomycosis, and clobetasol, used in psoriasis, were selected as model drugs. Optimised tattooing parameters enabled the lacquers to penetrate the microchannels created by the tattoo device, as confirmed by Raman spectroscopy and laser scanning confocal microscopy. Permeation studies demonstrated significantly greater diffusion of both ciclopirox and clobetasol in tattooed nails compared with filed or non-treated controls, while confocal imaging further verified the accelerated penetration of DexULac^®^ following tattoo application. Together, these findings highlight nail tattooing as a minimally invasive, point-of-care method for enhancing drug delivery through the nail, with strong potential for future clinical application in onychomycosis and psoriasis.

## Data Availability

All data supporting the findings of this study are available upon request from any of the authors.

## Declarations

### Supplementary Information

No gels or blots were generated or used in this study; therefore, uncropped gel or blot images are not applicable.
